# Reliability of central venous pressure to assess left ventricular preload for fluid resuscitation in patients with septic shock

**DOI:** 10.1186/s40560-014-0058-z

**Published:** 2014-10-10

**Authors:** Takako Sasai, Hiroaki Tokioka, Tomihiro Fukushima, Takeshi Mikane, Satoru Oku, Etsu Iwasaki, Mizue Ishii, Hideyuki Mieda, Tomoki Ishikawa, Eriko Minami

**Affiliations:** Department of Anesthesiology, Okayama Red Cross Hospital, 2-1-1 Aoe, Kita-ku, Okayama, Okayama 700-8607 Japan

**Keywords:** Septic shock, Central venous pressure, Echocardiography

## Abstract

**Background:**

Initial fluid resuscitation is an important hemodynamic therapy in patients with septic shock. The Surviving Sepsis Campaign Guidelines recommend fluid resuscitation with volume loading according to central venous pressure (CVP). However, patients with septic shock often develop a transient decrease in cardiac function; thus, it may be inappropriate to use CVP as a reliable marker for fluid management.

**Methods:**

We evaluated 40 adult patients with septic shock secondary to intra-abdominal infection who received active treatment and were monitored using transthoracic echocardiography (TTE) and CVP for 2 days after admission to our intensive care unit (ICU). We measured left ventricular end-diastolic diameter (LVEDD), left atrial diameter (LAD), and the pressure gradient of tricuspid regurgitation (TR∆P). The shock status was treated with volume loading and inotrope/vasopressor administration according to the TTE findings. We assessed left ventricular fractional shortening (LVFS) as an index of left ventricular contractility and TR∆P as an index of right ventricular afterload and then examined the correlation between CVP and LVEDD/LAD/TR∆P.

**Results:**

LVFS decreased to ≤30% in 42.5% and 27.5% of patients with septic shock, and severe left ventricular dysfunction with LVFS ≤20% developed in 12.5% and 15.0% of patients on the first and second ICU days, respectively, despite the use of inotropes/vasopressors. Mild pulmonary hypertension as indicated by TR∆P ≥30 mmHg was present in 27.5% and 30.0% of patients on their first and second ICU days, respectively. There was no significant correlation between CVP and LVEDD/LAD/TR∆P. The hospital mortality rate in this study was 10.0%, although the predicted mortality based on the Acute Physiology and Chronic Health Evaluation II score was 58.7%.

**Conclusions:**

Our results suggest that CVP is not a reliable marker of left ventricular preload for fluid management during the initial phase of septic shock. Assessment of left ventricular preload, right ventricular overload, and left ventricular contractility using TTE seems to be more informative than the measurement of CVP for fluid resuscitation since some patients developed left ventricular dysfunction and/or right ventricular overload.

## Background

Initial fluid resuscitation is an important hemodynamic therapy for patients with septic shock. The Surviving Sepsis Campaign Guidelines (SSCG) recommend the use of central venous pressure (CVP) as a marker of intravascular volume status [1]. Therefore, CVP has been traditionally used to assess fluid status; however, its value as a tool for guiding fluid resuscitation has been debated [2]. Patients with severe sepsis/septic shock often exhibit transient cardiovascular dysfunction [3]; thus, it may be inappropriate to use CVP as a reliable marker for fluid management. In addition, CVP, which is the pressure recorded in the superior/inferior vena cava near the right atrium, does not appear to be associated with intravascular volume or left ventricular preload. To investigate whether CVP is a reliable marker of left ventricular preload for fluid resuscitation in patients with septic shock, we assessed cardiac function using transthoracic echocardiography (TTE) and investigated the association of CVP with left ventricular end-diastolic diameter (LVEDD), left atrial diameter (LAD), and the pressure gradient of tricuspid regurgitation (TRΔP).

## Methods

This retrospective study was approved by The Okayama Red Cross Hospital Ethics Committee.

All adult patients with septic shock secondary to intra-abdominal infection who were admitted to the intensive care unit (ICU) between April 2006 and March 2012 were included in this study. Among them, patients who received active treatment, such as surgical removal of the source of infection, and who were monitored using TTE and CVP for 2 days after ICU admission were selected for analysis. We excluded patients who did not require norepinephrine support, lacked data of TTE monitoring on the first or second ICU day, developed septic shock after elective surgery, stayed in ICU <48 h, or had terminal cancer.

We measured LVEDD, left ventricular end-systolic diameter (LVESD), LAD, and TR∆P. Left ventricular fractional shortening (LVFS) was calculated by dividing the differences between LVEDD and LVESD by LVEDD.

We aimed to keep the mean arterial pressure at ≥80 mmHg and the urine output at ≥1 mL/kg/h. For fluid resuscitation, we aimed to maintain an intravascular volume with LVEDD 40–50 mm, LAD 25–35 mm, and TR∆P ≤30 mmHg, as evaluated by TTE. We used the following inotropes/vasopressors and diuretics: dopamine, ≤10 μg/kg/min; norepinephrine, ≤0.5 μg/kg/min; vasopressin, ≤0.017 U/min (1 U/h); furosemide, ≤10 mg/h; and carperitide, ≤0.69 μg/min (1,000 μg/day).

We assessed LVFS as an index of left ventricular contractility and TR∆P as an index of right ventricular afterload and then examined the correlation between CVP and LVEDD/LAD/TR∆P. Data of fluid intake, net output, and calculated fluid balance during the first 48 h were collected.

### Statistical analysis

Data are expressed as mean ± standard deviation or median (Q1–Q3; interquartile range). Statistical analysis was performed using Excel 2011 (Microsoft USA) with the add-in software Statcel 3 [4]. Data were analyzed using Pearson’s correlation coefficient or Spearman’s correlation coefficient by rank test depending on the distribution pattern. The median (95% confidence interval, CI) values of FS and TRΔP were analyzed by Wilcoxon’s signed-rank test. A *P* value of less than 0.05 was considered statistically significant.

## Results

**Figure 1:**
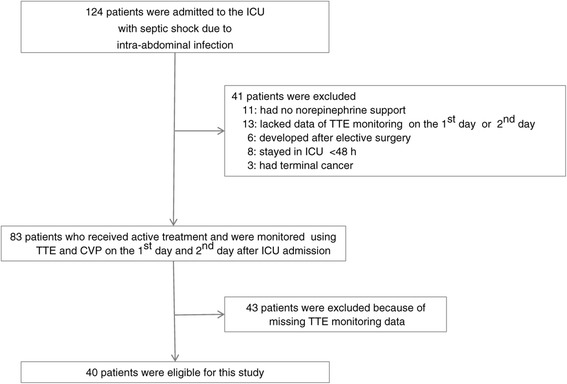
**Screening of study patients.**

The number of adult patients with septic shock secondary to intra-abdominal infection during the study term was 124. Among these, 40 patients (19 men and 21 women; mean age, 76.1 ± 10.4 years) were deemed eligible for participation in this study and were included in the final analysis (Figure 1). The most common source of sepsis was perforation of the lower digestive tract (Table 1). Thirteen patients had preexisting heart disease and five had preexisting respiratory disease (Table 2).

**Table 1:** **Source of sepsis of 40 patients**

**Source of sepsis**	**Number of patients**
Lower digestive tract perforation	13 (32.5%)
Liver/biliary tract disease	9 (22.5%)
Urinary tract infection	8 (20%)
Colitis	4 (10%)
Upper gastrointestinal tract perforation	3 (7.5%)
Ileus	2 (5%)
Non-obstructive intestinal ischemia	1 (2.5%)

**Table 2:** **Preexisting cardiopulmonary complications of 40 patients**

**Preexisting disease of 40 patients**	**Number of patients**
Heart disease	13 (33%)^a^
Ischemic heart disease	6
Chronic heart failure	3
Aortic stenosis	3
Atrial fibrillation	3
Mitral regurgitation	2
Pulmonary hypertension	2
Dilated cardiomyopathy	1
Sick sinus syndrome	1
Tricuspid regurgitation	1
Respiratory disease	5 (12.5%)
Bronchial asthma	2
Pulmonary aspergillosis	1
Bronchiectasis	1
Pneumoconiosis	1

^a^Six patients had two or three concomitant cardiac conditions.

**Figure 2:**
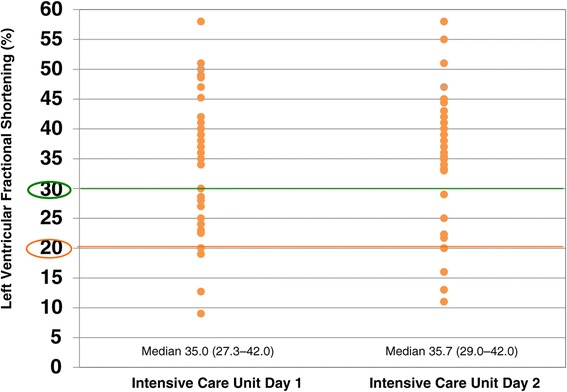
**Left ventricular fractional shortening in patients with septic shock.** Left ventricular fractional shortening decreased to ≤30% in 42.5% and 27.5%, and to ≤20% in 12.5% and 15.0% of patients on the first and second intensive care unit (ICU) days, respectively. There was no significant difference (*P* = 0.72) in median value between the first and second ICU days.

**Figure 3:**
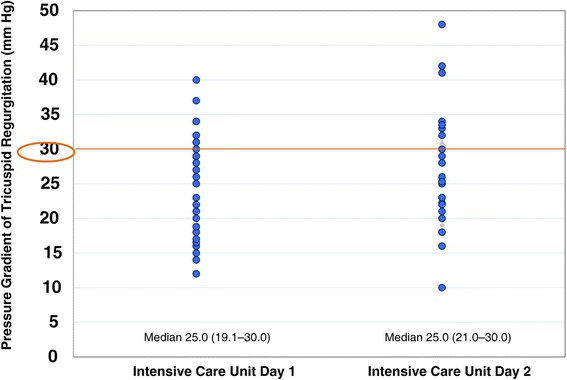
**Pressure gradient of tricuspid regurgitation in patients with septic shock.** Mild pulmonary hypertension with a pressure gradient of tricuspid regurgitation of ≥30 mmHg occurred in 27.5% and 30.0% on the first and second intensive care unit (ICU) days, respectively. There was no significant difference (*P* =0.17) in median value between the first and second ICU days.

**Figure 4:**
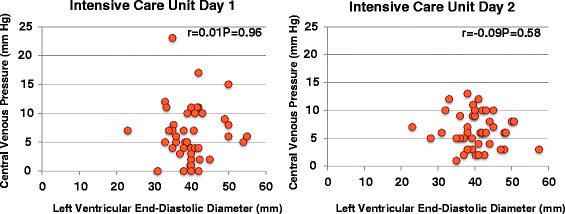
**Central venous pressure versus left ventricular end-diastolic diameter in patients with septic shock.** There was no significant correlation between central venous pressure and left ventricular end-diastolic diameter on the first and second intensive care unit days.

**Figure 5:**
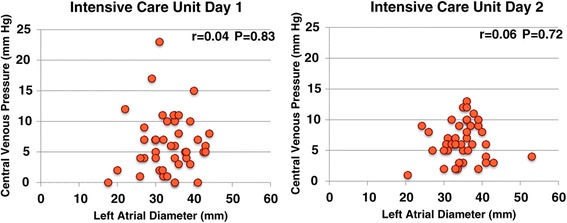
**Central venous pressure versus left atrial diameter in patients with septic shock.** There was no significant correlation between central venous pressure and left atrial diameter on the first and second intensive care unit days.

**Figure 6:**
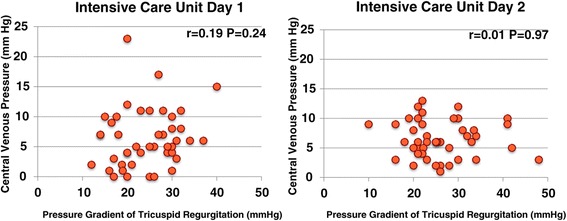
**Central venous pressure versus the pressure gradient of tricuspid regurgitation in patients with septic shock.** There was no significant correlation between central venous pressure and the pressure gradient of tricuspid regurgitation on the first and second intensive care unit days.

LVFS decreased to ≤30% in 17 (42.5%) and 11 (27.5%) patients on the first and second ICU days, and severe left ventricular dysfunction with LVFS ≤20% occurred in five (12.5%) and six (15.0%) patients on the first and second ICU days, respectively, despite the use of inotropes/vasopressors (Figure 2), and mild pulmonary hypertension with TR∆P ≥30 mmHg occurred in 11 (27.5%) and 12 (30.0%) patients on the first and second ICU days, respectively (Figure 3). There was no correlation between CVP and LVEDD on the first and second ICU days (correlation coefficients, 0.01 and −0.09, respectively; Figure 4). As shown in Figures 5 and 6, there were no correlations between CVP and LAD or TR∆P with correlation coefficients of 0.04 and 0.19 on the first ICU day and 0.06 and 0.01 on the second ICU day, respectively.

The median doses of intravenous fluid intake during the first and second 24-h periods were 5,216 (4,311–6,618) mL and 2,927 (2,480–3,566) mL, whereas net outputs were 3,280 (2,445–4,540) mL and 3,156 (2,510–4,040) mL, and fluid balances were 2,455 (1,051–3,528) mL and −71 (−853–287) mL, respectively.

The mean Acute Physiology and Chronic Health Evaluation (APACHE) II score was 24.9 ± 6.6. The ICU mortality rate was 2.5% (one patient) and the 28-day mortality rate was 5.0% (two patients). The in-hospital mortality rate was 10.0% (four patients), which was less than the predicted rate of 58.7% calculated from the APACHE II scores. The median ICU stay was 9.5 (6–21) days. Three patients required blood purification.

## Discussion

We used TTE to investigate whether CVP could be a useful guide for the assessment of left ventricular preload and volume status in the early phase of septic shock. Our results showed there was no association between CVP and LVEDD/LAD/TR∆P, suggesting that CVP is an inappropriate marker of left ventricular preload for fluid resuscitation.

CVP is currently the most practical target for fluid resuscitation and may be used to gauge fluid balance ≤12 h into septic shock, but it becomes unreliable as a marker of fluid balance thereafter [5]. Previous reports have suggested that CVP-guided fluid resuscitation may be inappropriate for septic shock [6–8]. In a systematic review by Marik et al. [6], they examined the relationship between intravascular volume and CVP in patients with sepsis and postoperative patients and determined the correlation coefficient of CVP with intravascular volume of 0.16, and the correlation coefficient of baseline CVP with a change in cardiac index was 0.18 and the area under the receiver operating characteristic curve was 0.56, indicating a poor correlation. Furthermore, a subsequent review reported similar results. The correlation coefficient between the baseline CVP and the change in cardiac index was 0.18 (95% CI, 0.1–0.25); therefore, these data do not support the widespread practice of using CVP as a guide for fluid therapy [7]. In addition, a study of healthy subjects determined that CVP was not useful to predict response to volume load because it did not reflect the ventricular filling volume or cardiac function [8].

In the early stages of sepsis, a high cardiac output (hyperdynamic) state was conventionally considered to be present; however, left ventricular contractility has been reported to be decreased following increased cardiac output. In the present study, left ventricular contractility was decreased on the first and second ICU days and severe left ventricular dysfunction (LVFS ≤20%) occurred in 13% and 15% of patients, and left ventricular dysfunction (LVFS ≤30%) was evident in 43% and 28%, respectively, despite the use of inotropes/vasopressors. Pulido et al. [9] reported the presence of myocardial dysfunction in 64% of patients with severe sepsis/septic shock within 24 h of ICU admission, with left ventricular systolic dysfunction and diastolic dysfunction present in 27% and 37% of patients, respectively.

With regard to right ventricular afterload, 28% of patients in this study presented with moderate pulmonary hypertension, with TR∆P ≥30 mmHg on the day of ICU admission. Right ventricular dysfunction also occurs in some patients without preexisting pulmonary hypertension [2]. Pulido et al. [9] described right heart failure in 31% of patients within 24 h of ICU admission. Baron et al. [10] also reported right heart failure in a case of septic cardiomyopathy. The SSCG indicate that CVP should not be used as a marker of intravascular volume status in patients with preexisting pulmonary hypertension.

The policy of early fluid resuscitation and inotrope/vasopressor use is an important factor influencing outcome in patients with septic shock. Fluid overload may trigger heart failure, potentially causing cardiac depression, especially in patients with septic cardiomyopathy. Extreme volume loading is associated with an increased risk of mortality [5]. In our series, four patients showed LVEDD ≥50 mm despite CVP ≤8 mmHg. In such patients, CVP-guided volume loading may cause heart failure. Actually, the infusion volume of these four patients was less than that of the others included in this study. Furthermore, the infusion volume in ICU is generally less than that reported by Boyd et al. [5]. Therefore, evaluation of cardiac function is important to guide fluid management in patients with septic shock. CVP is an unsuitable index in this regard. So, we usually evaluate left ventricular preload, right ventricular afterload, and left ventricular contractility using TTE and regulate volume loading with inotrope/vasopressor administration on the basis of the TTE findings instead of CVP.

TTE is a versatile, accurate, and noninvasive tool suitable for bedside examinations of patients with septic shock and has become a widely used hemodynamic-monitoring technique in ICU [11]. However, evaluation using echocardiography is a static measurement of a single time point that makes the evaluation of continuous parameters difficult. Accurate assessment of cardiac function and intravascular volume is possible by performing frequent measurements. We performed TTE frequently until the patient hemodynamics stabilized. Recently, various dynamic parameters (e.g., stroke volume variation, pulse pressure variation, global end-diastolic volume, and intrathoracic blood volume) have been proposed for the evaluation of fluid therapy and hemodynamics in patients with septic shock [12]. However, many of these parameters require dedicated equipment, whereas TTE does not require a special device.

Among our patients, the mean APACHE II score was 24.9 and the in-hospital mortality rate was 10.0%, which was much lower than the predicted rate of 58.7% calculated from the APACHE II scores. These results support the utility of TTE.

There are some limitations to this study that should be addressed. First, the study design was retrospective and thereby potentially subject to systematic error and bias, and the sample size was relatively small because of exclusion of cases with missing values. In addition, we were unable to fix specific time points for sampling; and therefore, these data may not be as precise as they could be. Hence, our results concern only the initial phase of septic shock. Second, there was no control group in which only CVP was used. Therefore, we were unable to definitely conclude that TTE is superior to CVP. Thus, further prospective studies with a control group in which only CVP was used are required to show the superiority of TTE. However, an assessment of the left ventricular preload, right ventricular overload, and left ventricular contractility using TTE was informative for fluid resuscitation. Third, we used different target values (mean arterial pressure and urine output) than those recommended by SSCG. This might have affected the outcome. We used a higher target mean arterial pressure because the patients admitted to our hospital with septic shock were often elderly and many had hypertension. Advanced age and chronic hypertension cause a rightward shift of the curve for organ pressure-flow autoregulation; and therefore, an increase in the mean arterial pressure could result in improved organ perfusion. Regarding the urine flow target, we used different criteria because the administration of transfusion or antimicrobial agents becomes difficult if the urine output is not maintained. Thus, we attempted to maintain the urine output in order to keep the treatment as conservative as possible.

## Conclusions

Our results showed that there was no correlation between the CVP and TTE findings in patients with septic shock. Therefore, CVP may not be a reliable marker of left ventricular preload for fluid management during the initial phase of septic shock. We observed that the assessment of left ventricular preload, right ventricular afterload, and left ventricular contractility using TTE is more informative than the measurement of CVP during initial fluid resuscitation in patients with septic shock, because patients with severe sepsis/septic shock often develop cardiovascular dysfunction.

## Availability of supporting data

The data sets supporting the results of this article are included within the article.

## Abbreviations

CVP: central venous pressure
TTE: transthoracic echocardiography
LVEDD: left ventricular end-diastolic diameter
LAD: left atrial diameter
TR∆P: pressure gradient of tricuspid regurgitation
ICU: intensive care unit
LVESD: left ventricular end-systolic diameter
LVFS: left ventricular fractional shortening
CI: confidence interval
APACHE: Acute Physiology and Chronic Health Evaluation
SSCG: Surviving Sepsis Campaign Guidelines
